# Research on the Industrial Energy Eco-Efficiency Evolution Characteristics of the Yangtze River Economic Belt in the Temporal and Spatial Dimension, China

**DOI:** 10.3390/ijerph17010268

**Published:** 2019-12-30

**Authors:** Zhonglin Tang, Geng Sun, Min Fu, Chuanhao Wen, Anđelka Plenković-Moraj

**Affiliations:** 1 Research Center for Economy of Upper Reaches of the Yangtze River & School of Economics, Chongqing Technology and Business University, Chongqing 400067, China; tangzl@ctbu.edu.cn; 2 China-Croatia “Belt and Road” Joint Laboratory on Biodiversity and Ecosystem Services, Chengdu Institute of Biology, Chinese Academy of Sciences, Chengdu 610041, China; 3 School of Intelligent Manufacturing, Chongqing Industry Polytechnic College, Chongqing 401120, China; fmsicnu@126.com; 4 School of Economics, Yunnan University, Kunming 650091, China; 5 Department of Biology, Faculty of Science, University of Zagreb, Rooseveltov trg 6, 10000 Zagreb, Croatia; aplenk@biol.pmf.hr

**Keywords:** Yangtze River Economic Belt, industrial energy eco-efficiency, spatial Markov chain

## Abstract

Based on the panel data of the 11 provinces along the Yangtze River Economic Belt from 1997 to 2015, the super slack-based model (Super-SBM) model is adopted to calculate the provincial-level eco-efficiency of industrial energy. While bringing in time series analysis and spatial differentiation feature analysis, the traditional and spatial Markov probability transition matrix is established. This study delves into the spatial-temporal dynamic evolution traits of the eco-efficiency of industrial energy along the Yangtze River Economic Belt. According to the results: the eco-efficiency of industrial energy of the Yangtze River Economic Belt manifests “single crest” evolution and distribution traits from left to right and top to bottom, indicating that the eco-efficiency of industrial energy of the Yangtze River Economic Belt is steadily improving gradually. However, the overall level is still low and there is still ample room for the improvement of the eco-efficiency of industrial energy. Furthermore, the eco-efficiency of industrial energy along the Yangtze River Economic Belt is elevating. The geographical spatial pattern plays a pivotal role in the spatial and temporal evolution of eco-efficiency of industrial energy, and the spatial agglomeration traits are noticeable.

## 1. Introduction

Striking a balance between industrial development and environmental protection, while improving the ecological efficiency (eco-efficiency, EE) in the process of development, has become an imperative issue that all countries, particularly developing countries, need to solve urgently [1,2,3,4]. Since Schaltegger and Sturm [5] put forth the concept of eco-efficiency as a “business link to sustainable development “in 1990, the definition of eco-efficiency has been further expanded by relevant organizations [6,7,8], such as the World Business Council For Sustainable Development (WBCSD), European Environment Agency (EEA), and the Organization for Economic cooperation and Development (OECD). Among them, the OECD’s definition has been widely accepted, which defines it as the efficiency of ecological resources to meet human needs [7,8]. The OECD has extended the term to government, industrial enterprises, and other organizations. Hitherto, eco-efficiency has been widely recognized and accepted by academia as well as the business sector, and has evolved into a significant indicator for the measurement of the coordinated development of the economy and environment [9,10,11,12,13]. Especially in recent years, with increasing attention drawn to global climate change and environmental issues, eco-efficiency studies have become popular in the field of research on sustainable development [9,13,14,15].

Current eco-efficiency research mainly focuses on application and calculation [16]. In terms of application, the existing research has discussed from the micro, meso, and macro levels, touching upon the construction of the indicator system and analysis of the influencing factors [17,18,19]. With regards to the calculation of eco-efficiency, due to differing research objects, spatial scales, and research objectives, the existing methods vary, chief among which are the single ratio method, the indicator system method, and the model method [20]. The single ratio evaluation method measures eco-efficiency by calculating a simple ratio, which is highly operable and relatively simple for understanding of the model, but cannot effectively distinguish the impact of different environments on eco-efficiency. This method only integrates various environmental impacts into a specific environmental impact value, leading to the distortion of reality to some extent. Compared with the single ratio method, the indicator system method can effectively distinguish the impact of different environments on eco-efficiency, yet it is difficult to exclude the subjective factors. The model method is the fastest growing and most reliable in the field of eco-efficiency evaluation [21,22,23,24]. The commonly used models include data envelopment analysis (DEA), super efficiency DEA, and three-stage DEA and slacks-based measure (SBM), which are all based on DEA. Among them, the SBM model incorporates negative externality into the model. This effectively addresses the problem of the relaxation of input and output, and has gradually become the mainstream model for measuring eco-efficiency [25,26,27,28].

As far as the research object of this study is concerned, some researchers have studied the eco-efficiency of the Yangtze River Economic Belt from different perspectives, such as financial agglomeration and industrial structure. He [16] expounded on the fact that there is a spatial agglomeration effect in eco-efficiency, and no spatial spillover effect, financial agglomeration has a limited influence over the improvement of eco-efficiency. In terms of industrial undertaking and transfer, Wu [29] found that undertaking industrial transfer does not hurt the overall eco-efficiency of the Yangtze River Economic Belt, but it does have an obvious negative impact on that of the middle reaches. Ren [30] divided the industrial eco-efficiency into three subsystems from the perspective of eco-efficiency of industrial energy, industrial economy, environment and energy, and conducted evaluation on the industrial eco-efficiency of nine provinces and two cities along the Yangtze River Economic Belt with the network DEA model. In general, research on the eco-efficiency of the Yangtze River Economic Belt is mainly focused on agriculture and the whole industry, while that on the industrial eco-efficiency of the Yangtze River Economic Belt is rare, especially on the industrial energy consumption of this area. Furthermore, the current research tends to adopt the radial DEA model for evaluation and does not take into account the undesirable output such as industrial wastewater and waste discharge in the industrial production process. Meantime, most of the research highlights the static time scale, while pays less attention to the description of the evolution characteristics of industrial eco-efficiency on the dynamic time and space scale. Therefore, this study looks into the industrial eco-efficiency in 1997–2015 by means of the SBM model and the spatial Markov model from the perspective of energy consumption. The time-varying and transposition characteristics on the spatial scale of industrial energy eco-efficiency are analyzed to clarify the temporal and spatial evolution processes of industrial energy eco-efficiency in the Yangtze River Economic Belt, and the impact of geographical pattern on industrial energy eco-efficiency. Thus, we hope to provide scientific support for further strengthening the cooperation of interregional industries and alleviating the imbalance of regional ecology in the Yangtze River Economic Belt.

## 2. Materials and Methods

### 2.1. Studied Area

**Figure 1:**
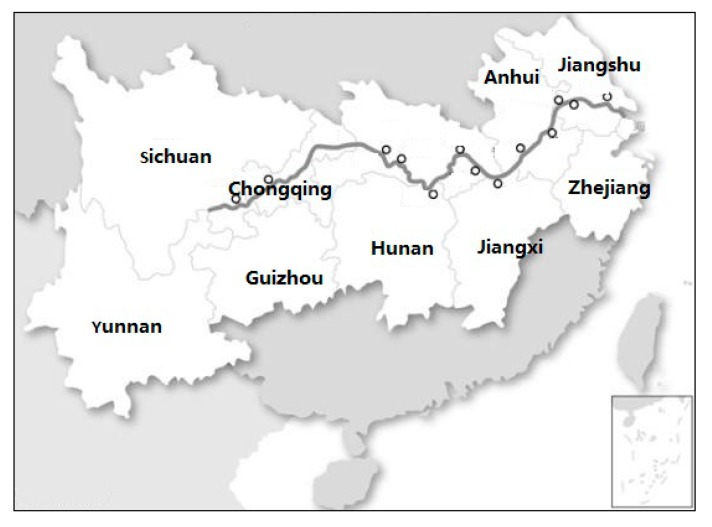
Location Map of the Yangtze River Economic Belt.

The Yangtze River Economic Belt covers 11 provinces and cities including Shanghai, Jiangsu, Zhejiang, Anhui, Jiangxi, Hubei, Hunan, Chongqing, Sichuan, Yunnan and Guizhou, covering an area of about 2,052,300 square kilometers (Figure 1), accounting for 21.4% of the country’s total, with the population and gross domestic product (GDP) exceeding 40% of the country’s total. As one of China’s major regional development strategies, the Yangtze River Economic Belt plays a vital supporting role in national economic growth and ecological security protection [31,32]. The priority given to environment protection and green development represents the guiding principle for the development of the Yangtze River Economic Belt.

Achieving a balance between national economic developments, the well-being of residents, and environment protection calls for the elevation of the existing eco-efficiency of industrial and agricultural production. For a long period of time, heavy and hazardous chemical industries have been sprawling along the Yangtze River Economic Belt, whose resource consumption and emissions rank among the top in China [33,34]. As of 2017, there were more than 400,000 chemical industrial enterprises along the Yangtze River Economic Belt, the sewage discharged by whom accounting for over 40% of the national aggregate [35]. In addition, the per unit area of ammonia-nitrogen and sulfur dioxide emitted was 1.5-times to twice the national average. While the industrial output value continues to rise, the environment surrounding the Yangtze River Economic Belt comes under daunting pressure and the ecological capacity is confronted with enormous challenges. Therefore, as an important approach to improving the quality of development, optimizing the efficiency of resource utilization and industrial structure is critical to the Yangtze River Economic Belt currently. Increasing the eco-efficiency of industrial input products has become a significant starting point to address the issue of sustainable development along the river [36]. Therefore, considering the current situation of industrial development and research status in the Yangtze River Economic Belt, this study focuses on the study of industrial energy consumption efficiency in the Yangtze River Economic Belt, and takes the unexpected output, such as industrial waste gas in industrial consumption into the super-SBM model to conduct the dynamic evaluation of eco-efficiency on a time scale. Meanwhile, the spatial Markov chain has been applied to explore the internal evolution of panel data on time and space scales. Combined with the traditional and spatial Markov probability transfer matrix, this study quantitatively depicts the spatiotemporal dynamic evolution characteristics of industrial energy eco-efficiency and its constraints on the geographical pattern in the Yangtze River Economic Belt, so as to provide scientific support for improving the eco-efficiency of industrial energy use and sustainable development of the Yangtze River Economic Belt.

### 2.2. Methods

#### 2.2.1. Super-SBM model Based on Undesirable Output

The CCR model (Named after A. Charnes & W. W. Cooper, and E. Rhodes), data envelopment analysis (DEA) model and super slack-based model (Super-SBM) model are widely used to measure eco-efficiency [13,21]. Among them, the CCR model based on constant return to scale means that every decision-making unit is in the optimal production scale state, but this is not in line with the actual situation. Most decision-making units are in the variable scale state. As the radial DEA model cannot measure the change of relaxation variables, the non-radial super-SBM model under variable return to scale is utilized in this study to measure the super efficiency of the Yangtze River. The formula of industrial energy eco-efficiency is as follows [36,37]:

$$\min\rho =\frac{1+\frac{1}{m}\sum_{i=1}^{m}s_{i}^{-}/x_{ik}}{1-\frac{1}{s}\sum_{r=1}^{s}s_{r}^{+}/y_{rk}}$$

$$s.t\sum_{j=1,j\neq k}^{n}x_{ij}\lambda_{j}-s_{i}^{-}\text{≤}x_{ik}(i=1,2,\ldots ,m)$$

$$\sum_{j=1,j\neq k}^{n}y_{rj}\lambda_{j}+s_{r}^{+}\text{≥}y_{rk}(r=1,2,\ldots ,s)$$

$$\lambda_{j}\text{≥}0,j=1,2,\ldots ,n(j\neq k),s_{i}^{-}\text{≥}0,s_{r}^{+}\text{≥}0$$

where ρ is the relative efficiency value, x and y are the input and output variables respectively, m and s are the numbers of indicators of input and output respectively, while $s_{i}^{-}$ and $s_{r}^{+}$ are the relaxation variables of input and output, respectively, and $\lambda_{j}$ is the weight vector. When ρ < 1, the decision-making unit is relatively ineffective, and when ρ ≥ 1, the decision-making unit is relatively effective.

#### 2.2.2. Non-Parametric Kernel Density Estimation

Kernel density estimation belongs to density mapping, which is more accurate and smoother than histogram estimation in describing the distribution of variables [23]. In essence, it is a process of surface interpolation through discrete sampling points, that is, with the smoothing method, using a continuous density curve instead of a histogram with its excellent statistical characteristic. Its basic principle is: Kernel density estimation, as a non-parametric estimation method, can use continuous density curve to describe the distribution form of random variables. Let the density function of random variables be *f*(*x*). For random variables *y*, there are *n* independent observations with the same distribution, which are *y*_1_, *y*_2_, …, *y*_n_, and the estimator of the Kernel density function is [38]:

$$f(x)=\frac{1}{nh}\sum_{i=1}^{n}K\left(\frac{y_{i}-y}{h}\right)$$

where n is the number of research areas; h is the width of the window; *K*(·) is a random kernel function, which is a weighted function or a smoothing function, including Gaussian (normal) kernel, Epanechnikov kernel, triangular kernel, and quartic kernel among other types. The selection of the window width determines the smoothness of the estimated density function. The larger the window width is, the smaller the variance of the kernel estimation is, the smoother the density function curve is, but the larger the deviation of the estimation. Therefore, selection of the optimal window width must be balanced between the variance and deviation of the kernel estimation to minimize the mean square error. The corresponding optimal window width is $h=cN^{-0.2}$ (*C* is constant). In this study, the kernel density function of Gaussian Kernel distribution is deployed, and the window width is set to be $h=0.9SeN^{-0.2}$ (i.e., C = 0.9se; Se is the standard deviation of the observation value of random variables).

#### 2.2.3. Spatial Markov Chain 

Markov chain is a statistical method based on the probability theory and the stochastic process theory, which uses the stochastic mathematical model to analyze the quantitative relationship in the development and change of real activities [39]. The traditional Markov chain is extracted according to Markov’s stochastic process theory. It measures the state of the event as well as its change and development trend by constructing the state transition probability matrix. In this process, given the current knowledge or information, the past (i.e., the historical state before the current period) is irrelevant to the prediction over the future (i.e., the future state after the current period), i.e., “no aftereffect”, also known as “Markovian”. As there is no aftereffect in the evolution process of many economic phenomena in reality, Markov chain is widely used in the field of economic management. The evolution process of industrial energy eco-efficiency studied in this study also has “no aftereffect”. The Spatial Markov chain analysis brings in the concept of spatial lag into the transfer probability matrix because the evolution of regional economic growth and other economic phenomena is not isolated and random in geographical locations, but closely intertwined and interacting with neighboring regions [39,40]. The spatial Markov chain makes up for the lack of spatial correlation of the research area in the traditional Markov chain analysis, which is used to shed light upon the correlation between the spatial and temporal evolution of an economic phenomenon and the regional spatial background.

### 2.3. Construction of the Indicator System

#### 2.3.1. Data Preparation

This study delves into the coordination of industrial and resource environment to calculate the industrial eco-efficiency, focusing on the eco-efficiency of energy consumption. The research objects are the 11 provinces (cities) in the Yangtze River Economic Belt. Chongqing, located in the upper reaches of the Yangtze River Economic Belt, was put directly under the Central Government as a municipality in 1997. As the statistical yearbook data related to the municipality directly under the Central Government was not systematically recorded until 1997, to measure the eco-efficiency of industrial energy in Chongqing, 1997–2015 is selected as the research period. The industrial data used in this study all come from the China Statistical Yearbook, China Environmental Statistical Yearbook, China Agricultural Yearbook, China land and Resources Statistical Yearbook and other yearbook data, as well as the websites of the National Bureau of Statistics and other relevant provincial and municipal bureaus of statistics along the Yangtze River Economic Belt.

#### 2.3.2. Indicator System

In effect, the eco-efficiency of industrial energy is established to realize the largest industrial economic output and ecological protection with the least industrial resource input and the lowest environmental cost. It reflects the win–win relationship between industrial economy, resource utilization and environmental protection in an all-dimensional way. The rate evaluation indicator system (Table 1), coupled with the reality and integrity of data acquisition, uses the number of industrial employees, industrial water, industrial power, industrial diesel, industrial gasoline, and industrial raw coal as the regional industrial resource input indicators, the total industrial output value as the expected output indicator, and the industrial waste discharge and industrial waste water discharge as the unexpected output, and constructs the Yangtze River Economic Belt indicator system of eco-efficiency of industrial energy.

The undesirable industrial output generally demonstrates the pollution caused by industrial development to the environment in terms of air, water, and soil. In this study, industrial waste gas emissions and industrial wastewater emissions are selected as the undesirable output. On the one hand, the actual situation of industrial pollution in the Yangtze River Economic Belt is taken into account; on the other, industrial waste gas and wastewater emissions can be more easily quantified, and the research period is also considered (1997–2015).

## 3. Results

### 3.1. Analysis on the Status Quo of Industrial Production and Development

**Figure 2:**
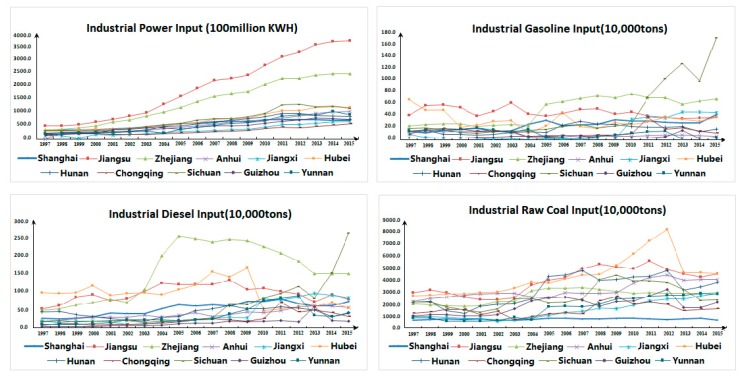
Industrial Input Diagram of the Yangtze River Economic Belt.

By comparing the input-output data of industrial production in provinces (cities) in the Yangtze River Economic Belt from 1997 to 2015, it can be found that: Seen from the input data, the overall final consumption of industrial power has been increasing, while the growth rate differs substantially (Figure 2). Among them, Jiangsu Province has the fastest growth rate, averaging annually at 2.81%. Zhejiang Province had the second highest, with an average annual growth rate of 2.58%. The final consumption of industrial gasoline presents an upward trend with fluctuations. The final consumption of industrial diesel in most provinces (cities) is on the rise, while that in Zhejiang, Sichuan, Hubei, and other provinces fluctuates significantly in the short term. The final consumption of industrial raw coal shows a rising trend first and then declines. 

**Figure 3:**
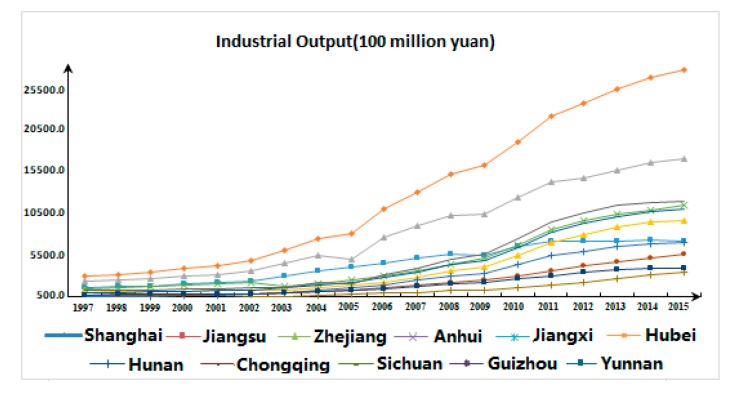
Industrial Output Diagram of the Yangtze River Economic Belt.

In terms of desirable output data (Figure 3), the industrial added value of the 11 provinces (cities) in the Yangtze River Economic Belt demonstrated a steady upward trend from 1997–2015, while those of Zhejiang Province and Sichuan Province dropped slightly from 2004–2005, and that of Shanghai also decreased moderately between 2008 and 2009. 

**Figure 4:**
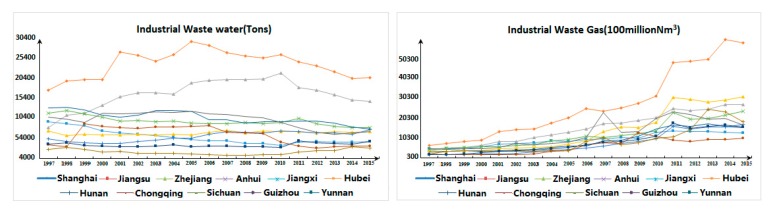
Industrial Undesirable Output Diagram of Yangtze River Economic Belt.

Concerning undesirable output data (Figure 4), most provinces (cities) in the Yangtze River Economic Belt displayed a reversed “U” trend of first fluctuating and then declining year by year from 1997 to 2015. For instance, Jiangsu Province and Zhejiang Province showed an upward trend of fluctuations from 1997 to 2010, while both declined year by year after 2010. As a whole, the consumption of electricity, gasoline and diesel in most provinces and cities of the Yangtze River Economic Belt rose from 1997 to 2015, but the consumption of raw coal decreased year by year. Furthermore, with the continuous increase of industrial added value, the discharge of industrial wastewater shows a reversed “U” trend with an initial rise followed by decline, which means that with the process of industrial modernization, the extensive industrialization mode of resource consumption has gradually evolved into the intensive industrialization mode of resource saving, but there is still ample room for industrial structural adjustment and optimization.

### 3.2. Measurement of Industrial Energy Eco- Efficiency

**Figure 5:**
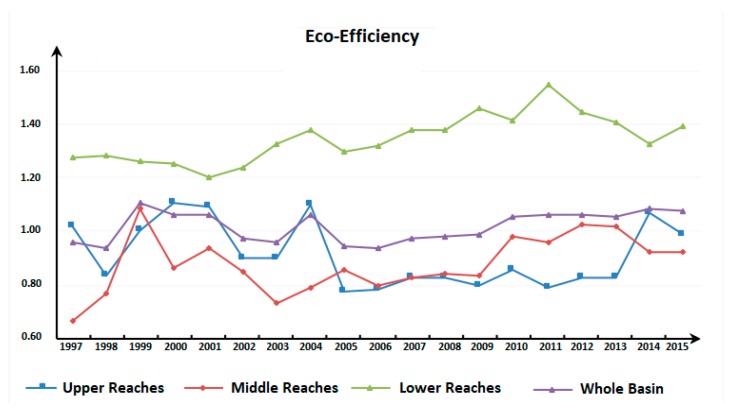
1997–2015 Evolution Trend of the Eco-efficiency of Industrial Energy in the Yangtze River Economic Belt.

Based on the DEA-SOLVER Pro 5.0(SAITECH, Inc. Holmdel, NJ, USA), with the non-oriented and variable return to scale (VRS) super-SBM model, this study estimates the eco-efficiency of industrial energy of the 11 provinces (cities) in the Yangtze River Economic Belt in China from 1997 to 2015, and carries out a comparative analysis on the average eco-efficiency of industrial energy of the upper, middle and lower reaches of the Yangtze River Economic Belt (Figure 5). By observing the trend in Figure 5, it can be noted that the eco-efficiency of industrial energy of the entire Yangtze River Economic Belt is basically at the level of 1.02 every year. In general, the eco-efficiency of industrial energy of the Yangtze River Economic Belt is at a low level, meaning there is sufficient room for the improvement of resource conservation and environmental protection in the pursuit of industrial development along the Yangtze River Economic Belt. From 1997 to 2015, the eco-efficiency of industrial energy of the Yangtze River Economic Belt shows a fluctuating uptrend, with the fluctuations occurring mainly between 1997 and 2015, which are noticeable. The 17th National Congress of the Communist Party of China proposed that the country’s mode of economic development should be changed. In the near future, the industrial economy will still be the mainstay of China. Efforts should be made to not only ensure the stable development of the industrial economy, but also to minimize the damage to the environment. This shows that the government attaches great importance to the sustainable development of industry in their avoidance of the decline of eco-efficiency of industrial energy.

**Figure 6:**
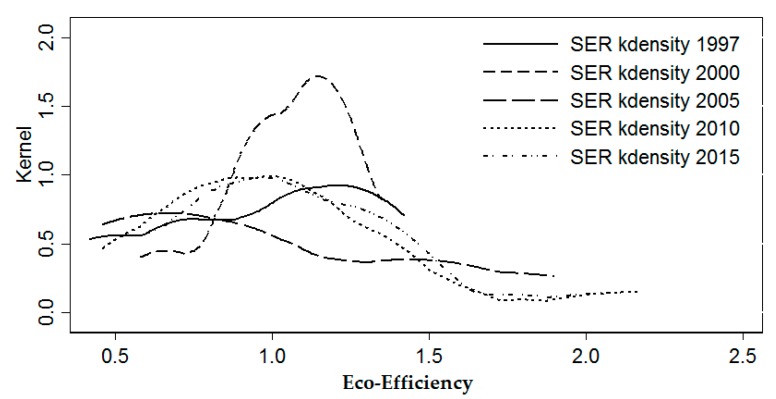
Kernel Density Estimation on the Eco-efficiency of Industrial Energy along the Yangtze River Economic Belt.

In order to continue exploring the agglomeration differences of the eco-efficiency of industrial energy of the 11 provinces (cities) in the Yangtze River Economic Belt over time, the non-parametric kernel density function of Gaussian normal distribution is adopted, and the years of 1997, 2000, 2005, 2010, and 2015 are selected to estimate the nuclear density. The distribution of different time points is observed (Figure 6). The height of the wave crests reflects the concentration of the eco-efficiency of industrial energy of each province (city). It can be concluded from Figure 6 that the overall eco-efficiency of industrial energy of the Yangtze River Economic Belt presents the evolution and distribution characteristics of "single crest" from left to right and the wave crests from high to low, which shows the trend that the eco-efficiency of industrial energy of the Yangtze River Economic Belt increases steadily gradually. Most provinces (cities) transform from a low level of agglomeration to no wave crests. In 1997, the eco-efficiency of industrial energy of most provinces (cities) in the Yangtze River Economic Belt gathered at a low level. After 2000, with the enhancement of industrial environmental protection awareness and the acceleration of industrial technological development, the eco-efficiency of industrial energy in the provinces (cities) in the Yangtze River Economic Belt has been improved to varying degrees, yet there are still differences in resource endowment, economic strength, etc. among the provinces (cities), and the eco-efficiency of industrial energy of the provinces (cities) is still different. The gap between eco-efficiency began to widen. In contrast, by 2015, there was no obvious crest distribution, indicating that the gap between low and high-level eco-efficiency of industrial energy was further narrowed.

### 3.3. Time Evolution Characteristics of the Eco-efficiency of Industrial Energy along the Yangtze River Economic Belt

Time analysis and kernel density analysis of the eco-efficiency of industrial energy can merely manifest the trend of time changes and evolution differences of the eco-efficiency of industrial energy, yet cannot profoundly reflect its internal space and time evolution law. First, the traditional Markov chain probability transfer matrix is deployed to divide the 11 provinces (cities) in the Yangtze River Economic Belt into different spaces per the differences of the eco-efficiency of industrial energy between 1997 and 2015. Given that the measurement of the provinces (cities) of each type is roughly the same, based on the method of quantile division, with 1/4, 1/2 and 3/4 quantiles as the boundaries, the eco-efficiency of industrial energy values are divided into four adjacent but not intersecting complete ranges: (0.31, 0.66), (0.66, 1.05), (1.15, 1.25), and (1.25, 2.49). The complete ranges are respectively represented by k = 1,2,3,4. The larger k is, the higher the regional eco-efficiency of industrial energy. Further, in accordance with the evolutional trend in Figure 6, the entire research period is roughly divided into two stages, namely 1997–2005 and 2006–2015. According to the classification of different types, the traditional Markov probability transfer matrix was obtained (Table 2).

The elements on the diagonal in Table 2 stand for the probability that the type of the eco-efficiency of industrial energy along the Yangtze River Economic Belt has not transferred, which reflects the stability of the evolution of the eco-efficiency of industrial energy of the Yangtze River Economic Belt, while the elements on the non-diagonal represent the probability that eco-efficiency of industrial energy of the Yangtze River Economic Belt has transferred between different state types. Therefore, it can be concluded that the evolution characteristics of the eco-efficiency without taking into consideration the geospatial pattern: (1) the eco-efficiency of industrial energy in all provinces (cities) in the Yangtze River Economic Belt is equipped with the stability of maintaining the original state. All the elements on the diagonal are significantly greater than those on the non-diagonals. The minimum value of the elements on the diagonal is 0.8074, and the maximum 0.8968. In other words, in whichever period, the eco-efficiency of industrial energy of a province (city) belongs to a certain type in a certain year, and the probability of belonging to that type in the following years is at least 80%. In addition, the probability of maintaining the stability of Type 4 is the largest from 1997 to 2015, and it is likely that the eco-efficiency of industrial energy converges for higher. (2) The eco-efficiency of industrial energy displays a salient trend of transferring to the high level, but the transfers in different stages vary. From 1997 to 2005, the probability gap between Type 2 and Type 3 is large, while that between P_22_ and P_33_ is also substantial, which indicates that the possibility of continuous improvement of eco-efficiency of industrial energy in each province (city) at this stage is greater, while between 2006 and 2015, P_21_ = 0.1447 > P_23_ = 0.0451, P_32_ = 0.0303 < P_34_ = 0.1091, demonstrating that the probability of the downturn of Type 2 is high, while that of the upturn of Type 3 is high. (3) It is difficult to realize the leapfrog transfer of the eco-efficiency of industrial energy. The transfer of the eco-efficiency of industrial energy almost occurs at one certain end of the diagonal. From the perspective of non-diagonal elements, the probability on the non-diagonal is significantly less than that on the diagonal, with a maximum value being 0.1658, merely 20.54% of the minimum probability on the diagonal. That means between two consecutive years, the probability of eco-efficiency of industrial energy to realize the leapfrog transfer is extremely small (e.g., 1 → 3), indicating that the improvement of eco-efficiency of industrial energy in various provinces and cities is a relatively stable and continuous process. It is difficult to achieve leapfrog development in the short term.

### 3.4. Spatial Evolution Characteristics of Eco-efficiency of Industrial Energy in the Yangtze River Economic Belt

This study depicts the spatial distribution map of the eco-efficiency of industrial energy type transfer in the two periods of 1997–2005 and 2006–2015 based on ArcGIS9.3, and the three periods of the former two in addition to 1997–2015. Figure 5 shows that from 1997 to 2015, the eco-efficiency of industrial energy of the Yangtze River Economic Belt shows an overall uptrend. Among them, ta 7 shows that the middle reaches of the region as a whole shows an upward growth trend. While in the upstream areas, except for Chongqing and Sichuan, a downward trend is spotted. Seen from the two periods of 1997–2005 and 2005–2015, the eco-efficiency of industrial energy in the middle reaches shows an obvious growth trend in different periods. Combined with the previous analysis of the results of the eco-efficiency of industrial energy, this is largely attributable to the lower level of eco-efficiency in the middle reaches, and also attests to the fact that the middle reaches have been paying greater attention to the coordination between industrial production, resource conservation, and environmental protection in recent years. In the upper reaches, except for Sichuan and Chongqing, the other two provinces both show a downward trend, but between 2005 and 2015, the eco-efficiency of industrial energy in Guizhou Province and Yunnan Province showed an upward trend thanks to a wide range of measures such as technological transformation and upgrading; in the downstream region, the distribution stability of different types of eco-efficiency of industrial energy is poor over the two different time periods with a downward trend observed. Nonetheless, by comparing the eco-efficiency value of each province, it is found that the change values tend to be smaller, for example, 0.01 in Shanghai. In general, this reflects that the spatial pattern of the change of eco-efficiency of industrial energy is closely linked with the economic strength and technologies of the province (city). Simultaneously, at different stages in the research, different provinces in the upstream and downstream areas display noticeable upward and downward differentiation, while the upward concentration trend in the middle reaches is evident, which, to a certain extent, shows that there is “club convergence” in the eco-efficiency of industrial energy in the Yangtze River Economic Belt from 1997 to 2015. 

According to the above research, it can be found that the transfer of industrial energy eco-efficiency in the Yangtze River Economic Belt is not isolated geographically but is closely linked with the geographical environment. In order to further explore the spatial differentiation law of industrial energy eco-efficiency in the provinces and cities along the Yangtze River Economic Belt, while carrying out the analysis of the traditional Markov probability transition matrix, this study brings in the spatial and geographical factors, sets up the spatial Markov probability transition matrix by using the type of spatial lag of provinces (cities) in the initial year and analyzes the impact of industrial energy eco-efficiency by the neighboring areas. Generally speaking, the spatial Markov probability transition matrix is based on the spatial lag types of different regions in the initial year. The division of the two periods in the above research shows that the type transfer of industrial energy eco-efficiency is certain unstable in terms of time, and there is “club convergence” effect in spatial distribution. Judging from that, this study establishes the spatial Markov probability transition matrix (Table 3) in 1997–2005 and 2006–2015, respectively, to measure the impact of the surrounding areas of different provinces (cities) on the eco-efficiency of industrial energy.

**Figure 7:**
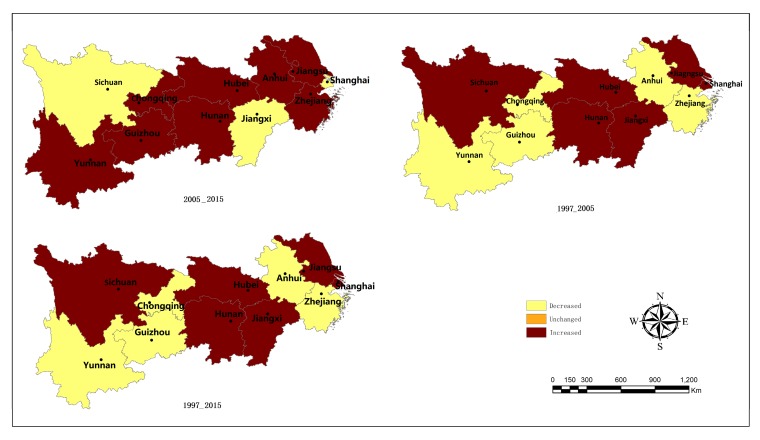
The change of Industrial Energy eco-efficiency in the Yangtze River Economic Belt.

Apart from the shared characteristics between the spatial Markov probability transition matrix and the traditional Markov probability transition matrix, by comparing the spatial distribution pattern of the two periods (Figure 7 and Table 3), it is found that when the eco-efficiency of industrial energy in a province (city) is transferred upward, the number of the surrounding provinces (cities) also augments, while that of the eco-efficiency of industrial energy in the surrounding provinces (cities) transferring downward is decreasing. Coupled with the results of the traditional Markov matrix analysis (Table 2), the following spatial evolution characteristics can be obtained: the geographical spatial pattern plays a vital role in the dynamic evolution of industrial energy eco-efficiency in the Yangtze River Economic Belt. In different neighboring backgrounds, the type transfer probability of industrial energy eco-efficiency of each province (city) differs and is not identical with the corresponding traditional Markov probability transition matrix. Or else, the impact of spatial lag will be invalid. For instance, without considering the geographical spatial pattern, P12 = 0.1658, while when a province (city) is adjacent to a Type 2 province (city), P12/2 = 0.1000 from 1997 to 2005. Hence, it is necessary to analyze the evolution and transfer of industrial energy eco-efficiency by taking the spatial background into account. Considering the influence of geographical spatial pattern, the probability of industrial energy eco-efficiency type transfer of a province (city) bordering on different types of provinces (cities) varies. In general, the probability of its upward transfer will increase if it is adjacent to the province (city) with higher industrial energy eco- efficiency, while the that of its downward transfer will increase if it is adjacent to the province (city) with lower industrial energy eco-efficiency. It can be seen that the provinces (cities) with high industrial energy eco-efficiency have a positive spillover effect on the neighboring provinces (cities), while those with low industrial energy eco- efficiency have no noticeable negative spillover effect on the neighboring provinces (cities). For example, during 2006 and 2015, the probability of upward transfer of a low-level (Type 1) area is P12 = 0.1393 (Table 2). When it is neighboring to a low-level (Type 2) area and a high-level (Type 3) area, the probability increases to 0.2353 and 0.5000 (Table 3) respectively. When it is next to a low-level area, the probability decreases to 0. The probability of an area transferring upward or downward is not proportional to the difference between a certain area and the neighboring areas. For a low-level area, should its adjacent area be of medium-low level, the probability of upward transfer between 2006 and 2015 is 0.2353 higher than when its adjacent area is of low level, and the probability of upward transfer is more transparent when its neighboring area is of medium-high level and high level, hitting 0.5 and 0.9091 respectively.

Using the comparative analysis of the aforementioned matrices, it can be found that the spatial background pattern has an important influence over the spatial and temporal evolution of the eco-efficiency of industrial energy in the Yangtze River Economic Belt. The provinces (cities) with high industrial energy eco-efficiency are more likely to boost the surrounding provinces (cities) to transfer upward, demonstrating a positive spillover effect. The probability of a region transferring up or down is not in proportion to the difference between of a certain area and the surrounding areas.

## 4. Discussion

This study deploys the panel data of the 11 provinces (cities) in the Yangtze River Economic Belt from 1997 to 2015, takes industry as the research object, and utilizes the super-SBM model to measure the inter-provincial eco-efficiency of industrial energy. With the time analysis based on kernel density estimation, this study sets up the traditional and spatial Markov probability transition matrix and explores the spatial and temporal evolution of eco-efficiency of industrial energy by analyzing the dynamic temporal and spatial evolution characteristics of industrial energy eco-efficiency through different matrices. 

Seen from the difference of time evolution, the eco-efficiency of industrial energy in the Yangtze River Economic Belt estimated via the broken line chart and Kernel Density Estimation displays an upward trend with fluctuations, which primarily concentrate from 1997–2005. During 2005 and 2015, the increase of eco-efficiency of industrial energy in the lower reaches is substantially higher than that of the upper and middle reaches, but the overall level is still low and there is ample room for improvement. The kernel density estimation chart shows that the evolution and distribution characteristics of "single peak" from left to right and wave crest from top to bottom attest to the fact that the eco-efficiency of industrial energy of the Yangtze River Economic Belt is steadily increasing with the passage of time. Currently, there is insufficient research on the industrial eco-efficiency along the Yangtze River Economic Belt and a lack of literature concerning the evaluation and comparison of the industrial energy eco-efficiency in that area. From the perspective of industrial energy consumption, this study looked into the industrial eco-efficiency in the Belt area and corroborated the conclusion drawn by Wu [29] and Ren [30], i.e., there is ample room for the improvement of resource conservation and pollution reduction in the area. Over the past decades, the industrial eco-efficiency in the area fluctuated significantly and there was substantial difference between the industrial eco-efficiency in the upper and lower reaches. 

At present, few studies have utilized the spatial Markov chain to study the spatial evolution of industrial energy eco-efficiency in the Yangtze River Economic Belt. Therefore, based on the spatial Markov chain, this study studies the spatial evolution of industrial energy eco-efficiency in the 11 provinces (cities) along the Yangtze River Economic Belt, and analyzes in depth the spatial evolution characteristics of industrial energy eco-efficiency in the Yangtze River Economic Belt, which is the extension and supplement of industrial eco-efficiency as well as industrial energy eco-efficiency research, and also provides more reliable empirical evidence for the improvement of the industrial energy eco-efficiency of the 11 provinces (cities) in the Yangtze River Economic Belt. In terms of the spatial evolution pattern, the industrial energy eco-efficiency in the Yangtze River Economic Belt demonstrates a noticeable “club convergence” effect regarding spatial distribution, and there is spatial dependence between the provincial industrial energy eco-efficiency. According to the traditional Markov probability transition matrix, the overarching trend of industrial energy eco-efficiency transfer to a high level is transparent, and the evolution displays the stability of maintaining the original state. Contrast conducted between the spatial Markov probability transition matrix and the traditional transition matrix shows that, beside the common characteristics, the geographical spatial pattern plays a significant role in the spatial-temporal evolution of industrial energy eco-efficiency, and the spatial positive spillover effect is noteworthy. Under different spatial backgrounds, the probability of industrial energy eco-efficiency transfer in different provinces and cities differs. If a certain province or city borders on another with high industrial energy eco-efficiency, the probability of its upward transfer increases, whereas if it is adjacent to another with low industrial energy eco-efficiency, the probability of downward transfer decreases.

In this study, the Markov probability transfer matrix is applied to study on the spatial-temporal evolution characteristics of the industrial energy eco-efficiency. The conclusions of this study are similar to the research on the pollution-intensive industries in the Yangtze River Economic Belt conducted by Li [41], meaning the distribution of the said industries is closely related to the geographical pattern, the hot spot areas of those industries are stable comparatively and the “club convergence” effect of their development is noticeable. Likewise, the dynamic research undertaken by Chen [42] on the pollutant emissions in the Yangtze River Economic Belt shows the internal mobility of pollutant emissions in cities with high and low pollution is low. There is a trend of club convergence and the odds of transfers towards the neighboring types are high. The results underscored not only the intertwined correlation of the efficiency in the subsequent years, but also the impact of geographical layout on the evolution process of the industrial energy eco-efficiency. That shows incorporating the spatial lag into the construction of the measurement model for the calculation of influencing factors causing damage to the industrial energy eco-efficiency is necessary.

Furthermore, on the basis of the spatial and temporal evolution characteristics of industrial energy eco-efficiency in the Yangtze River Economic Belt derived by the super-SBM model and the spatial Markov probability transition matrix, the following implications can be drawn:

The super-SBM model based on undesirable output can take the impact of resources and environment on the industrial energy eco-efficiency into full account and can carry out further comparison on the DMU with the efficiency of 1. Nevertheless, this study fails to incorporate various social and economic factors that lead to the loss of industrial energy eco-efficiency. Since the loss is caused by a multitude of factors, the future research can build in factors such as structural change, policy change, technological change, urbanization, foreign direct investment (FDI), etc., and delve into the influencing factors that result in the loss of industrial energy eco-efficiency.

The spatial Markov probability transition matrix is one of the effective ways to study the temporal and spatial evolution characteristics of different economic phenomena (industrial energy eco-efficiency). We do not just highlight the intertwined relationship between the years before and after the research object, but also emphasize the influence of geographical spatial pattern on the evolution process of industrial energy eco-efficiency. In addition, this study includes the spatial lag condition in the measurement model for the influencing factors of the loss of industrial energy eco-efficiency to set up the measurement model of spatial panel. This heralds the developmental trend of future research.

## 5. Conclusions

Based on the super-SBM model, this study calculates the eco-efficiency of industrial energy in the Yangtze River Economic Belt from 1997 to 2015 and constructs the traditional and spatial Markov probability transfer matrix on the basis of time series analysis and spatial differentiation feature analysis. The results show that: The eco-efficiency of industrial energy in the Yangtze River Economic Belt has the characteristics of “single peak” evolution and distribution from left to right and top to bottom, which indicates that the eco-efficiency of industrial energy in the Yangtze River Economic Belt has the trend of stable improvement with the change of time, but it is still at a low level as a whole, and there is still ample room for the improvement of the eco-efficiency of industrial energy. Furthermore, in general, the eco-efficiency of industrial energy along the Yangtze River Economic Belt is improving. The geographical spatial pattern plays a pivotal role in the spatial and temporal evolution of eco-efficiency of industrial energy, and the spatial agglomeration traits are noticeable.

In summary, this study is far from being complete. On the one hand, in choosing the indicators of industrial energy input, output, and unexpected output, it establishes an indicator system based on the actual situation of the Yangtze River Economic Belt, but it is difficult to carry out a horizontal comparison with the other research for want of unified standards. On the other hand, this study focuses on the temporal changes, spatial layout, and consequences of the industrial energy eco-efficiency in the Yangtze River Economic Belt, while failing to discuss the influencing factors of the aforementioned efficiency and its impact on the environment and human beings. This should be consolidated in the follow-up research.

## Figures and Tables

**Table 1:** Measurement Indicator System of the Eco-efficiency of Industrial Energy in the Yangtze River Economic Belt.

Indicators	Variables	Breakdown of the Indicators	Unit
Industrial Input	Industrial Employees Industrial Water Industrial Power	Industrial Labor Input Amount of Industrial Water Input Industrial Power Input	10,000 persons 10,000 m^3^ 100 million KWH
	Industrial Diesel	Industrial Diesel Energy Consumption	10,000 tons
	Gasoline	Industrial Gasoline Energy Consumption	10,000 tons
	Raw Coal	Industrial Raw Coal Consumption	10,000 tons
Undesirable Output	Industrial Waste Gas	Exhaust Emissions Caused by Industrial Production and Development	100 million Nm^3^
	Industrial Wastewater	Wastewater Discharged by Industrial Production	Ton
Desirable Output	Industrial Output	Industrial Output Value	100 million yuan

**Table 2:** Traditional Markov Probability Transfer Matrix of Eco-efficiency of Industrial Energy in the Yangtze River Economic Belt from 1997 to 2015.

	1997–2005					2006–2015			
	1	2	3	4		1	2	3	4
1	0.8074	0.1658	0.0268	0	1	0.8117	0.1393	0.0489	0
2	0.0731	0.8968	0.0302	0	2	0.1447	0.8102	0.0451	0
3	0	0.1168	0.8145	0.0687	3	0	0.0303	0.8605	0.1091
4	0	0.0674	0.1142	0.8183	4	0	0.0538	0.1317	0.8146

**Table 3:** Markov Probability Transfer Matrix of Eco-efficiency of Industrial Energy in the Yangtze River Economic Belt from 1997 to 2015.

Spatial Lag	t/t+1	1997–2005				2006–2015			
		1	2	3	4	1	2	3	4
1	1	0	0	0	0	0	0	0	0
	2	0	1	0	0	0	0	1	0
	3	0	0	0	0	0	1	0	0
	4	0	0	0	1	0	0.3333	0	0.6667
2	1	0.7500	0.1000	0.1500	0	0.5882	0.2353	0.1765	0
	2	0.2222	0.5556	0.2222	0	0.2500	0.5000	0.2500	0
	3	0.1176	0.1765	0.5294	0.1765	0.0769	0.2308	0.6154	0.0769
	4	0	0.0833	0.2500	0.6667	0	0	0.2000	0.8000
3	1	0.6000	0.4000	0	0	0.5000	0.5000	0	0
	2	0.4545	0.4545	0.0909	0	0.4000	0.6000	0	0
	3	0	0.0909	0.8182	0.0909	0	0	1	0
	4	0	0	0.0833	0.9167	0	0	0	1
4	1	0.5000	0.5000	0	0	0	0	0	0
	2	0	1	0	0	0	0.9091	0	0.0909
	3	0.5000	0	0.5000	0	0	0	1	0
	4	0	0	0	1	0	0	0.0909	0.9091
